# Treatment Options for Pancreatic Neuroendocrine Tumors

**DOI:** 10.3390/cancers11060828

**Published:** 2019-06-14

**Authors:** Amit Akirov, Vincent Larouche, Sameerah Alshehri, Sylvia L. Asa, Shereen Ezzat

**Affiliations:** 1 Institute of Endocrinology, Beilinson Hospital, Petach Tikva 49100, Israel; 2 Sackler School of Medicine, Tel Aviv University, Tel Aviv 69978, Israel; 3 Department of Endocrine Oncology, Princess Margaret Cancer Centre, Toronto, ON M5G 1Z5, Canada; vincent.larouche@uhn.ca (V.L.); sameerah.alsherhri@uhn.ca (S.A.); Shereen.Ezzat@sinaihealthsystem.ca (S.E.); 4 Department of Medicine, Division of Endocrinology and Metabolism, Jewish General Hospital, McGill University, Montreal, QC H3T 1E2, Canada; 5 Department of Pathology, University Health Network, University of Toronto, Toronto, ON M5S 1A1, Canada; pathlady01@gmail.com

**Keywords:** neuroendocrine tumors, pancreas, pancreatic cancer

## Abstract

The management of pancreatic neuroendocrine tumors (PanNETs) involves classification into non-functional or functional PanNET, and as localized or metastatic PanNET. In addition, while most PanNETs are sporadic, these endocrine neoplasms can also be manifestations of genetic syndromes. All these factors may assist in forming a risk stratification system permitting a tailored management approach. Most PanNETs are classified as non-functional because they are not associated with clinical sequelae of hormone excess. They are characterized by non-specific symptoms, such as abdominal pain or weight loss, resulting from mass effect related to the pancreatic tumor or secondary to distant metastases. Accurate staging of the disease is essential for determining the appropriate approach to therapy. As cure is only potentially possible with surgical resection of the tumor, it is recommended to remove all localized and limited metastatic disease. However, many patients present with metastatic and/or advanced local disease. In such instances, the goal of therapy is to control tumor growth and/or decrease tumor burden, lengthen survival, and palliate local symptoms and those of hormone excess. This typically requires a multimodal approach, including surgery, liver-directed treatment, and systemic medical therapy.

## 1. Introduction

Neuroendocrine tumors can arise from a variety of different organs, including the endocrine tissue of the pancreas leading to pancreatic neuroendocrine tumors (PanNETs). While PanNETs are rare overall, with an annual incidence of less than 1 case per 100,000 individuals, these tumors are increasingly more common in the world [1]. These tumors are usually sporadic, but occasionally may be associated with germ-line mutation causing multiple hereditary endocrinopathies, such as multiple endocrine neoplasia type 1 (MEN1) or type 4 (MEN4), von Hippel-Lindau (VHL) disease, neurofibromatosis type I (NF1), and tuberous sclerosis [2]; more recent data also show associations with Lynch syndrome, and SDH-related familial paraganglioma syndromes.

The clinical presentation can be due to symptoms related to hormone over-secretion by the tumor, such as excess insulin (insulinoma), gastrin (gastrinoma), glucagon (glucagonoma), vasoactive intestinal peptide (VIPoma), somatostatin (somatostatinoma), or pancreatic polypeptide (PPoma). While non-functioning PanNETs can secrete several peptides, such as chromogranin, the symptoms in these cases are mainly secondary to local mass effect of the tumor, such as abdominal pain, weight loss, nausea, and less frequently, obstructive jaundice or hemorrhage [2].

The prognosis of a PanNET depends on clinico-pathological factors, such as tumor size, proliferative index, and differentiation [3]. The WHO classification of PanNETs is based on Ki-67 expression and mitotic counts. Grades 1 and 2 are considered well-differentiated tumors (Ki-67 < 20%), while grade 3 tumors are further classified to well-differentiated NET or neuroendocrine carcinoma (NEC) [4]. In addition, genetic mutations may also have prognostic significance [3].

Accurate staging of the disease is essential for determining the appropriate approach to therapy. As cure is only potentially possible with surgical resection of the tumor, it is recommended to remove all localized and limited metastatic disease. However, many patients present with metastatic and/or advanced local disease. In such instances, the goal of therapy is to control tumor growth and/or decrease tumor burden, lengthen survival, and palliate local symptoms and those of hormone excess. This typically requires a multimodal approach, including surgery, liver-directed treatment, and systemic medical therapy.

## 2. Surgical Treatment

### 2.1. Surgically Resectable Disease

**Figure 1:**
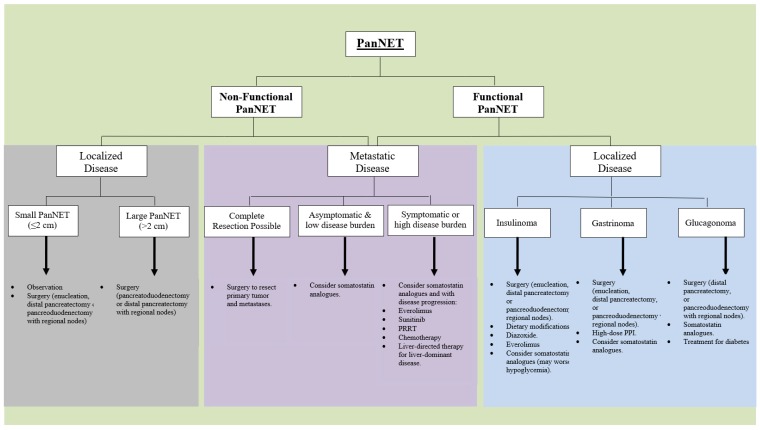
Treatment Algorithm. Suggested treatment algorithm for pancreatic neuroendocrine tumors, based on functionality and localized or metastatic disease.

Surgery is the only treatment approach that can cure a PanNET. In addition, surgical resection may be used to alleviate symptoms secondary to local mass effect or excess hormone levels [2,5]. In patients with localized PanNET, the typical recommendation is surgical resection of the tumor wherever technically feasible (Figure 1). Exceptions may be due to severe comorbidities or high surgical risk, widely metastatic disease, or very small and sporadic non-functional PanNETs.

When surgery is indicated, a decision regarding the extent of surgery must be made. A complete surgical resection includes removing the primary tumor and any lymph node metastases. There are several pancreatic procedures to address a PanNET. (a) Enucleation: This approach is usually reserved for selected cases, usually small, functional PanNETs in the head of the pancreas; (b) distal pancreatectomy: This includes removal of the pancreatic body and tail, with or without splenectomy; (c) pancreaticoduodenectomy: This approach is common for a PanNET in the pancreatic head, which is not suitable for enucleation. In some instances, more extensive surgery is required, i.e., total pancreatectomy, while in others, a more targeted approach may be used, such as parenchyma-sparing resection. Resection of the tumor should include adequate regional lymph node resection. 

Several studies investigated the outcomes of non-operative management and reported that for non-functioning PanNETs, which are sporadic, asymptomatic, and ≤2 cm, a conservative approach with active surveillance is safe, as in most cases, there was no significant change in the size of the tumors during follow-up [6,7]. However, the guidelines are controversial whether surgery should be recommended as primary treatment for a PanNET ≤2 cm [8,9]. The NCCN guidelines recommend in cases of non-functional PanNETs >2 cm, invasive, or node-positive tumors, to complete pancreatoduodenectomy and regional nodes for tumors situated in the head of the pancreas, or distal pancreatectomy with splenectomy and regional nodes, for tumors in the distal pancreas [10]. For patients with functional tumors, the surgical approach may be more important. For instance, with gastrin- or insulin-secreting tumors originating in the pancreas, the NCCN recommend enucleation of the tumor with periduodenal node dissection for exophytic or peripheral tumors in the head of the pancreas and pancreato-duodenectomy for those deeper, more invasive, or close to the main pancreatic duct. Enucleation with peripancreatic lymph node dissection may also be considered for a small (<2 cm), peripheral glucagonoma, or VIPoma [10]. Distal pancreatectomy with regional nodes is recommended for tumors in the distal pancreas [10]. Similarly, the European Neuroendocrine Tumor Society (ENET) guidelines recommend that all patients with sporadic gastrinoma and without contraindication should have surgical exploration, with removal of peritumoral lymph nodes [8]. These guidelines also recommend surgical exploration in all patients with non-resectable metastatic insulinoma, with or without multiple endocrine neoplasia (MEN)1 [8].

As in the setting of MEN1, the tumors occur at an earlier age, and frequently present as multiple tumors, therefore the surgical approach is different compared to sporadic tumors. In cases of functional PanNETs other than gastrinoma, resection will usually be used for alleviation of symptoms secondary to hormone overproduction. There is controversy regarding the surgical treatment of multifocal small gastrinomas and non-functional PanNETs in MEN1, with no generally accepted guidelines.

### 2.2. Surgical Treatment for Advanced Disease

Tumor debulking is not curative and the main advantage of this approach is for symptom control in functional tumors and to prolong survival in functional and non-functional PanNETs. However, the data are not clear regarding this matter. Most guidelines agree that the surgical approach will be based on the extent of tumor burden. The NCCN guidelines recommend that the management of locoregional advanced disease and/or distant metastases, resection of the primary tumor, and the metastases be performed when complete resection is possible, and to consider non-curative debulking in selected cases [10]. Partial hepatectomy can be considered in patients with liver metastases.

When performing surgery for advanced PanNETs, it is recommended to consider cholecystectomy in patients anticipated to receive long-term octreotide therapy, due to the increased risk of biliary disease associated with somatostatin analogue therapy.

### 2.3. Post-Surgery Follow-Up

Following surgical resection of a PanNET, it is recommended to continue clinical, imaging, and biochemical follow-up, as clinically indicated. The clinical symptoms in patients with PanNETs depend on the functionality of the tumor, and can be very severe, depending on the hormone secreted. In all patients, chromogranin A may serve as a tumor marker, but one has to keep in mind that proton pump inhibitors, which are commonly used, may cause false elevation in chromogranin A (and gastrin) levels. Insulin, pro-insulin, and C-peptide will be used for those with insulinoma; pancreatic polypeptide (PP), VIP, glucagon, and gastrin, respectively, will be used for patients with a diagnosis of PPoma, VIPoma, glucagonoma, or gastrinoma. Imaging studies can include abdominal CT or MRI and chest CT, as clinically indicated. Somatostatin receptor-based imaging (68 Ga-dotatate PET/CT or somatostatin-receptor scintigraphy) is additionally available.

## 3. Medical Therapy

Medical treatment is required in many patients with advanced, recurrent, or metastatic PanNETs that are not candidates for surgical intervention (Figure 1). This medical treatment may be used to control hormone excess and alleviate symptoms, but can also be used to control the tumor growth. Of note, subtotal resection of a large proportion of the tumor can be considered in patients with symptomatic recurrence, while incomplete resection of advanced disease in patients with asymptomatic or non-functional disease is controversial [10].

### 3.1. Symptoms-Directed Therapy

The first consideration in patients with unresectable, functional PanNET, is hormonal control, as hormone overproduction may cause significant morbidity and mortality. Short- and long-acting somatostatin analogues may be used to alleviate symptoms of functional tumors, such as diarrhea and flushing that can be caused by various hormones. Other specific measures are as follows:

Gastrinoma—high doses of proton pump inhibitors are required for most patients with gastrinoma with acid hyper-secretion. Patients should usually be started on omeprazole 60 mg daily, esomeprazole 120 mg daily, lansoprazole 45 mg daily, rabeprazole 60 mg daily, or pantoprazole 120 mg daily, with dose titration as needed. Another option is an H2 receptor blocker. Somatostatin analogues may aid in controlling the associated diarrhea. Of note, proton pump inhibitors may lead to increased levels of chromogranin A and gastrin. Secretin test cannot be used while a patient is taking these medications, as they can also result in a false positive test. On the other hand, if proton pump inhibitors are abruptly stopped in patients with gastrinoma, complications related to high acid levels may develop rapidly.

Insulinoma—nutritional adjustment with frequent small meals, as well as suppression of insulin secretion with diazoxide, are frequently used for insulinoma. Somatostatin analogues can be used cautiously for glucose control, but as these may paradoxically lead to more frequent hypoglycemic events due to possible suppression of glucagon, it may be preferable to initiate treatment with short-acting octreotide, followed by a long-acting preparation, based on the clinical response. Everolimus has been associated with increased glucose levels in these patients and may serve as an important therapeutic option. 

Glucagonoma—in these patients, it is advised to consider parenteral nutrition, including vitamin supplementation. In addition, anti-coagulation is an important consideration due to the increased risk of thromboembolism, especially pre-operatively.

VIPoma—hydration, somatostatin analogues, and glucose control are all important factors in the symptomatic management of these patients.

Somatostatinoma—measures to relieve symptoms are important. Patients may require hydration and parenteral supplementation, as well as medication to control diabetes and pancreatic enzyme supplementation to reduce diarrhea and steatorrhea. Somatostatin analogues may be effective at controlling the symptoms secondary to excess hormone levels, although the effects may be less dramatic than for other functional PanNETs.

PPoma—in early stages, symptoms might not be evident as pancreatic polypeptide levels are elevated but no clinical syndrome is present. When symptoms occur, they may include abdominal pain and occasional watery diarrhea.

Carcinoid syndrome—telotristat ethyl is a tryptophan hydroxylase inhibitor that was found in the TELESTAR study to significantly decrease the frequency of bowel movements and urinary 5-hydroxyindole acetic acid in patients with serotonin-producing neuroendocrine tumors not adequately controlled on somatostatin analogues [11]. The treatment was approved by the FDA in February 2017 for persistent diarrhea in patients with carcinoid syndrome, which is unusual in patients with PanNETs that rarely secrete serotonin.

### 3.2. Control of Tumor Growth

There are three main different groups of medical therapies available for control of tumor growth: Somatostatin analogues (octreotide, lanreotide), molecular targeted treatment (everolimus, sunitinib, others), and chemotherapy (capecitabine, 5-fluorouracil, capecitabine, dacarbazine, oxaliplatin, streptozotocin, and temozolomide).

#### 3.2.1. Somatostatin Analogues

Somatostatin is a peptide that inhibits the secretion of a wide range of hormones, and somatostatin analogues (e.g., octreotide and lanreotide) have similar effects. As somatostatin receptors are usually abundantly expressed in neuroendocrine tumors, somatostatin will bind to those receptors, mainly somatostatin receptor type 2, thus inhibiting hormone secretion from the tumor cells. Somatostatin analogues may be effective in both controlling the symptoms of functional PanNETs, especially VIPomas and glucagonomas, with a lesser effect in patients with somatostatinomas or gastrinomas. As noted, in patients with insulinoma, the response may be unpredictable. In addition to controlling symptoms secondary to hormone over-production, these agents may control tumor growth [12]. The anti-proliferative efficacy of somatostatin analogue treatment in PanNETs was confirmed in the CLARINET trial, published in 2014 [12]. This was a phase III, multinational, randomized, placebo-controlled, double-blind study, involving 204 patients with well-differentiated neuroendocrine tumors (Ki67 antigen < 10%), originating in the pancreas, midgut, hindgut, or from unknown origin, with documented disease progression. All patients had avid disease on somatostatin-receptor scintigraphy. Treatment with lanreotide (Somatuline) was associated with a significant prolonged progression-free survival, compared with placebo (progression-free survival at 24 months: 65.1% with lanreotide (95% CI, 54.0–75.1 months) and 33.0% with placebo (95% CI, 23.0–43.3 months). However, the study did not prove a significant difference in overall survival between the active treatment and placebo group. Based upon these results, lanreotide has been approved in the United States for patients with gastroenteropancreatic neuroendocrine tumors.

Given the fact that many PanNETs may have an indolent disease course, the optimal time to initiate somatostatin analogue treatment in asympotomatic patients is unclear. The guidelines by the ENETS [13], NCCN [10], and North American Neuroendocrine Society (NANETS) [14] suggest initiation of somatostatin analog treatment in asymptomatic patients with high burden of well-differentiated, unresectable PanNET.

While this treatment is usually well-tolerated, common adverse effects of somatostatin analogue therapy may include gastrointestinal disturbances, mostly nausea, bloating, flatulence, diarrhea, and/or steatorrhea secondary to fat malabsorption (~30% of patients), but these tend to subside over time [12]. Up to a quarter of the patients may develop cholelithiasis or biliary sludge with this line of therapy, and for that reason, prophylactic cholecystectomy is recommended when performing surgery for advanced PanNET in patients anticipated to receive treatment with somatostatin analogues [10]. Mild glucose intolerance may also occur with somatostatin analogues.

#### 3.2.2. Molecularly Targeted Therapies

Two agents have been shown to improve progression-free survival in patients with advanced PanNET—everolimus and sunitnib. Other agents have also been used for control of tumor growth.

##### Everolimus

Everolimus (Afinitor), an oral a mammalian target of rapamycin (mTOR) inhibitor, is a common second-line treatment for patients with progressive metastatic PanNET (Figure 1). In the RADIANT-3 trial, a prospective, randomized, phase 3 study, 410 patients with advanced low- or intermediate-grade PanNET with documented disease progression were treated with everolimus or placebo, in combination with best supportive care. Treatment with everolimus was found to be superior to placebo, and prolonged median progression-free survival (11.0 vs. 4.6 months, *p* < 0.05) [15].

The common adverse effect of hyperglycemia associated with everolimus may be of particular value in patients with functioning insulinoma and refractory hypoglycemia. The frequency of grade 3/4 hyperglycemia is much higher in those with pre-existing diabetes mellitus. This treatment has also been associated with pneumonitis, stomatitis, dyslipidemia, peripheral edema, increased blood pressure, headache, insomnia, constipation/diarrhea, or neuromuscular pain [15,16,17].

##### Sunitinib

Sunitinib is an oral multi-targeted tyrosine kinase inhibitor, which was approved for the treatment of progressive well-differentiated PanNET in patients with unresectable, locally advanced, or metastatic disease. A multinational, randomized, placebo-controlled, double-blind, phase 3 trial reported improved progression-free survival (median progression-free survival was 11.4 vs. 5.5 months) and overall survival (mortality rate was 10% vs. 25%) with sunitinib, compared to placebo, in patients with advanced well-differentiated PanNETs. Due to this highly significant effect, trial accrual was stopped prematurely prior to the first preplanned interim efficacy analysis [18].

The data regarding the effect of sunitinib on symptoms of hormone over-production are limited. While there are some reports that this treatment may aid in the treatment of VIPomas, in those with insulinoma, this line of therapy may aggravate hypoglycemia [19,20,21].

Side effects associated with sunitinib may include elevated blood pressure, thyroid dysfunction, renal toxicity, arterial thromboembolism, heart failure, myelosuppression, hand-foot skin reaction, delayed wound healing, muscle wasting, and hepatotoxicity [18]. 

##### Pazopanib, Sorafenib, Surafitinib, and Axitinib

Pazopanib, Sorafenib, and Axitinib are multi-targeted kinase inhibitors that have also been examined in the treatment of NETs. Pazopanib inhibits VEGF receptors 1, 2, and 3, and an early study has shown it to have some effect in patients with PanNET, but not those with small bowel NETs [22]. Surufatinib, a small-molecule inhibitor targeting VEGF receptors, fibroblast growth factor receptor 1, and colony-stimulating factor 1 receptor showed promising anti-tumor activity in patients with advanced neuroendocrine tumors, including PanNETs [23].

##### Temisirolimus and Bevacizumab

The combination of temisirolimus, another mTOR inhibitor, with bevacizumab, a VEGF inhibitor, was studied in a multi-center phase II study of patients with locally advanced or metastatic, well-, or moderately differentiated PanNETs, with evidence of progressive disease. The combination treatment had substantial activity with a response rate of 41% (23 of 56 patients), and progression-free survival at six months was 79% [24]. The combination of bevacizumab and everolimus also demonstrated anti-tumor activity in patients with low- to intermediate-grade NET, and this combination was well tolerated [25].

In a large randomized trial comparing bevacizumab to interferon in 427 patients with metastatic gastroenteropancreatic or lung NET already on octreotide, radiologic response rates were more frequent among patients treated with bevacizumab than interferon (12% vs. 4%), but there was no difference in progression-free survival [26]. 

#### 3.2.3. Cytotoxic Chemotherapy

There is no consensus on the best cytotoxic chemotherapeutic regimen, and the possible agents in patients with symptomatic, and/or progressive disease include 5-FU, capecitabine, dacarbazine, oxaliplatin, streptozocin, and temozolomide. Frequently, a combination treatment will be preferred, and this can include temozolomide-capecitabine, 5-FU/doxorubicin/streptozocin (FAS), or streptozocin with either doxorubicin or 5-FU. 

##### Streptozocin-Based Chemotherapy

Streptozocin, like dacarbazine, is an alkylating agent, and studies have shown that the combination of streptozocin and doxorubicin is superior to streptozocin plus 5-FU in patients with advanced PanNET [27]. Combination of 5-FU, doxorubicin, and streptozocin (FAS) showed response rate of 39% in patients with locally advanced or metastatic PanNET, with longer progression-free and overall survival [28].

The BETTER trial showed that bevacizumab with 5-FU/streptozocin in patients with progressive metastatic well-differentiated PanNET reported progression-free survival of 23.7 months and overall survival at 24 months of 88% [29]. The possible adverse effects related to streptozocin include renal toxicity, which is dose-related and cumulative and may be severe or fatal, as well as nausea and vomiting, diarrhea, liver toxicity, and hematologic disturbances. 

##### Temozolomide-Based Chemotherapy

Temozolomide-based therapy has similar overall response rate to streptozocin-based therapy in patients with malignant PanNETs and represents an alternative to the latter. Temozolomide has been used as a monotherapy, or in combination with other agents, including bevacizumab and capecitabine, and it seems that temozolomide-based therapy may be most effective in patients with Ki-67 between 20–55% [30]. In 2011, a retrospective study showed that the combination of capecitabine and temozolomide (CAPTEM) was associated with a 70% objective response rate, with a median progression-free survival of 18 months and an overall survival of 92% at two years [31]. Recently, it was reported that progression-free survival in advanced PanNETs improved significantly with the addition of capecitabine to temozolomide in a randomized trial. The phase II trial ended after a planned interim analysis showed that the combination treatment met the primary endpoint, as median progression-free survival improved from 14.4 months with temozolomide alone to 22.7 months with the combination CAPTEM. A preliminary analysis of overall survival also showed a significant improvement with the combined therapy, although the median value had yet to be reached in the combined treatment group [32]. Common significant adverse events occurred twice as often in the combination arm (44% vs. 22%), and the most common events were neutropenia, thrombocytopenia, nausea/vomiting, diarrhea, fatigue, and lymphopenia. 

##### Platinum-Based Chemotherapy

Well-differentiated PanNETs are separated into low-, intermediate-, or high-grade (G1–G3). While initially it was thought that G3 tumors are poorly differentiated, it has become clear that some of the tumors in this category are relatively well-differentiated on histology and have a better prognosis, compared to the poorly differentiated tumors [33]. G3 tumors that are relatively well-differentiated histologically have relatively poor response to platinum-based chemotherapy. In contrast, patients with poorly differentiated tumors, known as neuroendocrine carcinoma (NEC), which frequently have a rapidly progressive clinical course, are candidates for combination chemotherapy with cisplatin and etoposide, or its analogs, with an estimated 40–70% response rate, albeit with a short duration of response [13,14].

## 4. Radiation Therapy

### 4.1. External Beam Radiotherapy (EBRT)

The role of external beam radiotherapy for patient with PanNETs is limited. There are limited, retrospective data on the overall activity and safety of EBRT in PanNETs and further studies are required [34].

### 4.2. Peptide Receptor Radionuclide Therapy (PRRT)

Peptide receptor radionuclide therapy (PRRT) involves delivery of targeted radiotherapy to malignant neuroendocrine tumor cells that express somatostatin receptors to cause tumor shrinkage. In 2017, the randomized-controlled NETTER-1 trial [35] reported better progression-free survival with ^177^Lu-Dotatate and Octreotide LAR, compared to Octreotide LAR alone in patients with well-differentiated metastatic midgut NET (progression-free survival at 20 months: 65.2% vs. 10.8%). In addition to the improved tumor growth, in the planned interim analysis of overall survival, 14 deaths occurred in the ^177^Lu-Dotatate group and 26 in the control group (*p* = 0.004). Potential rare toxicities from administration of ^177^Lu-Dotatate include myelosuppression and nephrotoxicity. However, that study did not include patients with PanNETs. Several other studies have reported high tumor control rates following administration of PRRT in patients with PanNETs [36,37,38,39,40]. Recently, the safety and efficacy of PRRT was investigated in 34 patients with metastatic functioning PanNETs (G1–G2), including insulinomas, VIPomas, gastrinomas, and glucagonomas. The treatment resulted in partial or complete response in 59% of patients, disease control in 78%, and reduction of symptoms in more than 80%. While the treatment was safe, the authors recommended considering prevention of hormonal crises, including high-dose proton-pump inhibitors for those with gastrinoma, fluids, anti-diarrheal medications, and short-acting octreotide for VIPomas, and glucose infusion or short-acting octreotide for insulinomas [41].

## 5. Liver-Directed Therapies

In patients with hepatic-dominant disease, liver-directed therapies may be considered. The treatment options include surgical resection, ablation (radiofrequency ablation [RFA], cryoablation, or microwave ablation), hepatic arterial embolization, chemoembolization, or radioembolization. In patients with functional PanNETs, all liver-directed therapies require the prior initiation of somatostatin analogues, or other specific symptom-controlling measures. Locoregional therapies are recommended early, following somatostatin analogue treatment, and these can be an alternative option to systemic therapies in cases of disease limited to the liver.

Surgical resection of liver metastases is an option for patients without diffuse bilobar involvement, liver dysfunction, extrahepatic metastases, or high-grade neuroendocrine carcinoma. In patients with functional PanNETs and liver metastases, surgery may be a valid option if the major tumor burden is located in the liver [42]. Historically, the recommendation for surgery was if at least 90% of the tumor can be removed. However, there are no randomized-controlled trials comparing liver resection vs. other approaches. Debulking surgery may be recommended in selective patients with liver-predominant functional PanNETs, even if the intervention can lead to <90% reduction of the liver tumor [42].

Ablation is usually reserved for small neuroendocrine liver metastases (<3 cm) in patients who are not candidates for surgery. As in many cases, liver-disease is multifocal and bilateral, ablation is usually used in addition to surgical resection, to avoid extensive hepatectomy. Hepatic artery embolization, chemoembolization, or radioembolization may be used for symptom control, while the survival benefit of these interventions is less clear. These techniques are especially important for patients with PanNETs in which the hormone excess cannot be controlled by other measures [14,43]. There are limited data comparing the various embolization techniques; thus, the optimal approach is unclear. Liver-directed therapies are associated with increased risk of perihepatic sepsis and liver abscess [44].

In highly selected patients, usually young patients with functional PanNET with no response to medical treatment, liver transplantation can be considered. However, orthotopic liver transplantation has been attempted in a small number of patients, and cannot be routinely recommended at this time.

## 6. Future Directions

Recent well-designed randomized controlled trials in the field of neuroendocrine tumors have led to significant changes in our understanding and treatment options for patients with these tumors. However, as our understanding of the molecular basis for these tumors has advanced, it has become clear that it is important to conduct further clinical trials that will distinguish PanNETs from other gastrointestinal neuroendocrine tumors, and well-differentiated PanNETs from poorly differentiated NECs. It will also be critical to investigate the long-term survival benefits of various treatment options.

Treatment with PRRT is expected to become more popular for patients with PanNETs, as outcome data will most likely support this treatment modality. While today this treatment is used mainly in the presence of disease progression following failure of previous therapy, it is possible that PRRT will be used more often and at an early course in appropriate patients. Furthermore, it is expected that in the future, treatment for PanNETs will most likely be tailored individually for each patient, based on the tumor biology, molecular, and genetic patterns. One possibility might include sequencing individual patient tumors and selecting the treatment based on mutations, such as treating with everolimus in cases with known mTOR mutations.

## 7. Conclusions

Surgery is the only treatment that can cure functional or non-functional PanNETs and in many cases this will be the first modality applied in these patients. For patients with non-functioning, small, sporadic, and asymptomatic PanNETs mainly in the head of the pancreas, a conservative approach with active surveillance is feasible, though this is controversial. Surgery may be more important in functional PanNETs, including those with metastases, as surgical debulking can be used to alleviate symptoms secondary to hormone excess.

In many patients with advanced or metastatic disease, medical treatment is required. This additional treatment may be directed to control the symptoms and/or tumor growth. Somatostatin analogues are frequently used as first-line treatment of unresectable PanNETs (Figure 1). For functional PanNETs, more specific modalities may be used, such as proton pump inhibitors for gastrinomas, or nutritional guidance and diazoxide for patients with insulinoma. Molecularly targeted therapies, specifically everolimus and sunitinib, may be used as second-line treatment, following disease progression with somatostatin analogues (Figure 1). However, for hepatic-dominant disease, liver-directed therapies may be considered at an early stage. Peptide receptor radionuclide therapy may be used in cases of more extensive disease, with extrahepatic metastases, and this approach can lead to improved survival. For G3 tumors that are well-differentiated, cytotoxic chemotherapy may be used; while there is no consensus on the best cytotoxic chemotherapy regimen, the combination of capecitabine and temozolomide (CAPTEM) was recently reported to significantly improve overall and progression-free survival. For patients with neuroendocrine carcinoma (NEC), which is poorly differentiated and frequently has a rapidly progressive clinical course, combination chemotherapy of cisplatin and etoposide, or its analogs, is recommended.
